# Clinically relevant body composition phenotypes are associated with distinct circulating cytokine and metabolomic milieus in epithelial ovarian cancer patients

**DOI:** 10.3389/fimmu.2024.1419257

**Published:** 2024-11-07

**Authors:** Evan W. Davis, Hua-Hsin Hsiao, Nancy Barone, Spencer Rosario, Rikki Cannioto

**Affiliations:** ^1^ Department of Biostatistics and Bioinformatics, Roswell Park Comprehensive Cancer Center, Buffalo, NY, United States; ^2^ Department of Cancer Prevention and Control, Roswell Park Comprehensive Cancer Center, Buffalo, NY, United States; ^3^ Department of Pharmacology and Therapeutics, Roswell Park Comprehensive Cancer Center, Buffalo, NY, United States

**Keywords:** body composition, adipose tissue, skeletal muscle, epithelial ovarian cancer, metabolomics, cytokines, immune suppression, tumor progression

## Abstract

**Introduction:**

Preclinical evidence suggests that host obesity is associated with tumor progression due to immuno-metabolic dysfunction, but the impact of obesity on immunity and clinical outcomes in patients is poorly understood, with some studies suggesting an obesity paradox. We recently reported that high-adiposity and low-muscle body composition phenotypes are associated with striking increases in epithelial ovarian cancer (EOC) mortality and we observed no evidence of an obesity paradox. However, whether at-risk versus optimal body composition phenotypes are associated with distinct immuno-metabolic milieus remains a fundamental gap in knowledge. Herein, we defined differentially abundant circulating immuno-metabolic biomarkers according to body composition phenotypes in EOC.

**Methods:**

Muscle and adiposity cross-sectional area (cm^2^) was assessed using CT images from 200 EOC patients in The Body Composition and Epithelial Ovarian Cancer Survival Study at Roswell Park. Adiposity was dichotomized as low versus high; patients with skeletal muscle index (SMI) <38.5 (muscle cm^2^/height m^2^) were classified as low SMI (sarcopenia). Joint-exposure phenotypes were categorized as: Fit (normal SMI/low-adiposity), Overweight/Obese (normal SMI/high-adiposity), Sarcopenia/Obese (low SMI/high adiposity), and Sarcopenia/Cachexia (low SMI/low-adiposity). Treatment-naïve serum samples were assessed using Biocrates MxP Quant 500 for targeted metabolomics and commercially available Luminex kits for adipokines and Th1/Th2 cytokines. Limma moderated T-tests were used to identify differentially abundant metabolites and cytokines according to body composition phenotypes.

**Results:**

Patients with ‘risk’ phenotypes had significantly increased abundance of metabolites and cytokines that were unique according to body composition phenotype. Specifically, the metabolites and cytokines in increased abundance in the at-risk phenotypes are implicated in immune suppression and tumor progression. Conversely, increased abundance of lauric acid, IL-1β, and IL-2 in the Fit phenotype was observed, which have been previously implicated in tumor suppression and anti-tumor immunity.

**Conclusion:**

In this pilot study, we identified several significantly differentially abundant metabolites according to body composition phenotypes, confirming that clinically significant joint-exposure body composition phenotypes are also biologically distinct. Although we observed evidence that at-risk phenotypes were associated with increased abundance of immuno-metabolic biomarkers indicated in immune suppression, additional confirmatory studies focused on defining the link between body composition and immune cell composition and spatial relationships in the EOC tumor microenvironment are warranted.

## 1 Introduction

Preclinical evidence across a variety of tumor models has established that host obesity is associated with tumor progression as a result of immune and metabolic dysregulation (1–9), but the impact of obesity on immunity and outcomes in patients remains an area of intense scientific debate and clinical interest, with many studies suggesting obesity is linked with improved immunotherapy and survival outcomes (an obesity paradox) (1, 2, 10–21). For instance, in epithelial ovarian cancer (EOC), the most recent meta-analyses summarizing the associations of obesity at diagnosis with mortality suggests there is either no association of excess adiposity with EOC mortality (15) or there is an obesity paradox (11).

For several years, our group has rigorously studied the relationships of body composition with EOC mortality and we recently reported novel data demonstrating that high-adiposity and low-muscle joint-exposure body composition phenotypes before chemotherapy are associated with striking increases in EOC mortality (22). For example, in comparison to the ‘Fit’ body composition phenotype (normal muscle mass and low adiposity), the ‘Overweight/Obese’ phenotype (normal muscle/high adiposity) was associated with up to 104% increased mortality; the ‘Sarcopenia/Obese’ phenotype (low muscle/high adiposity) was associated with 67% increased mortality; and the ‘Sarcopenia/Cachexia’ phenotype (low muscle/low adiposity) was associated with 109% increased EOC mortality (22). Hence, we showed that appropriately accounting for low muscle mass in joint-exposure analyses eliminates any evidence of an obesity paradox. However, what remains unknown is whether these four body composition phenotypes are associated with distinct immune and metabolic circulating milieus. To address this gap in knowledge, we initiated a pilot study to define differentially abundant cytokines and metabolites according to four clinically relevant joint-exposure body composition phenotypes in EOC patients diagnosed and treated at Roswell Park. Associations were defined in EOC overall and in patients diagnosed with high-grade serous ovarian carcinoma (HGSOC), the most common and deadly EOC subtype.

## 2 Methods

### 2.1 Study population

We leveraged data and specimens from women in The Body Composition and Epithelial Ovarian Cancer Survival (BComES) Study at Roswell Park Comprehensive Cancer Center in Buffalo NY (PI: Cannioto). The BComES Study is a survival cohort comprised of 750 pathologically confirmed invasive EOC patients treated at Roswell Park between 2006 and 2024. For the current study, we identified a sub cohort of EOC patients in the BComES study who also: 1) were consented and enrolled into the Data Bank and BioRepository (DBBR) at Roswell Park, a Comprehensive Cancer Center Shared Resource; 2) had treatment-naïve banked serum samples; 3) completed first-line treatment without significant treatment delays between 2006-2021; 4) had clinically measured height and weight recorded in the EHR; and 5) had a high-quality computed tomography (CT) image available in the Picture Archiving and Communication System. A total of 200 participants met the inclusion criteria and are included the current analysis. Approval to initiate the BComES Study was obtained in June 2019 from the Roswell Park Institutional Review Board; additional approval to initiate the current biomarker study was obtained in May 2022.

### 2.2 Body composition assessment and parameterization

Previous CT image-based body composition validation studies have established that cross-sectional body composition at the third lumbar vertebra (L3) is most representative of whole-body adiposity and muscle mass (23). Herein, standard-of-care CT images at L3 were manually segmented by a trained rater using a well-established standardized pipeline for analysis. We used commercially available, validated sliceOmatic software to segment and quantify the cross sectional area (cm^2^) at L3 for skeletal muscle, subcutaneous adipose tissue, intermuscular adipose tissue, and visceral adipose tissue (24).

Total adipose tissue (TAT) area was calculated as the sum of subcutaneous, intermuscular, and visceral adipose tissue area. In accordance with guidance established in the extant literature, a skeletal muscle index (SMI) was calculated as the ratio of skeletal muscle area (cm^2^) to patient height (m^2^); patients with SMI≥38.5 cm^2^/m^2^ were classified as having a normal SMI and patients with SMI<38.5 cm^2^/m^2^ were classified as having a low SMI, a proxy for sarcopenia (25). As no standard cut-points for adiposity at L3 exist, and tertile one coincides with the approximate percentage of normal-weight patients in our cohort according to body mass index (BMI), we used the cut-point for the lowest tertile of adiposity to classify low versus high adiposity.

**Figure 1:**
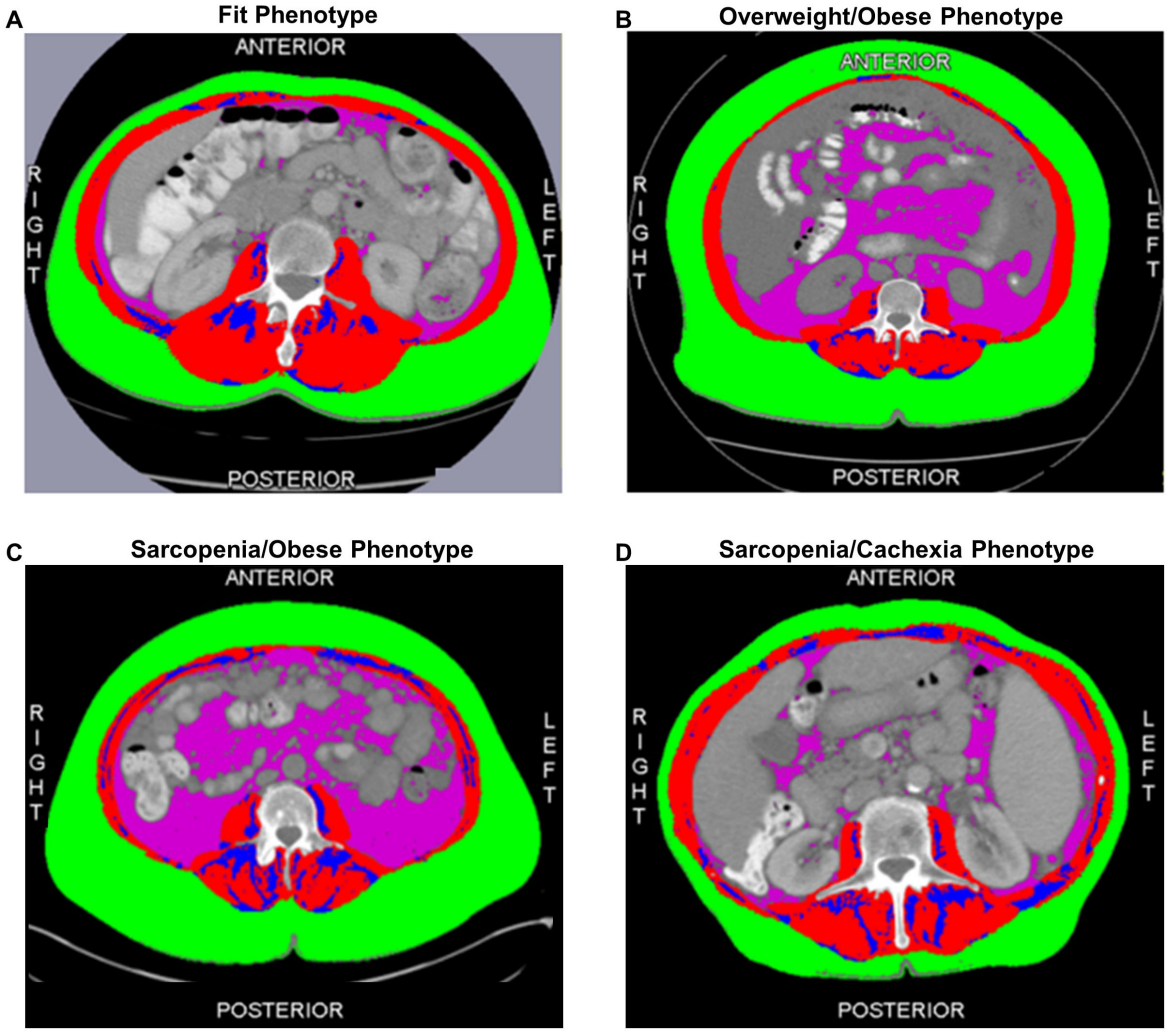
Cross-sectional computed tomography images at the third lumber vertebra depicting the adiposity and muscle distribution of each of the four body composition phenotypes including the **(A)** Fit (normal muscle/low total adiposity) Phenotype; **(B)** Overweight/Obese (normal muscle/high total adiposity) Phenotype; **(C)** Sarcopenia/Obese (low muscle/high total adiposity) Phenotype; and **(D)** Sarcopenia/Cachexia (low muscle/low total adiposity) Phenotype. The red compartment represents skeletal muscle; the green compartment represents subcutaneous adipose tissue; the blue compartment represents intermuscular adipose tissue; and the pink compartment represents visceral adipose tissue. Total adiposity is derived as the sum of the green, blue, and pink compartments.

Based on our previous reports (22, 26), our primary exposure of interest for the current analysis is a joint-exposure muscle and adiposity body composition phenotype. Representative cross-sectional CT images of each body composition phenotype are depicted in **Figure 1**. Specifically, circulating metabolites and cytokines will be defined according to four clinically relevant body composition phenotypes classified as: the Fit (referent) phenotype (normal SMI/low TAT) (**Figure 1A**); the Overweight/Obese phenotype (normal SMI/high TAT) (**Figure 1B**); the Sarcopenia/Obese phenotype (low SMI/high TAT) (**Figure 1C**); and the Sarcopenia/Cachexia phenotype (low SMI/low TAT) (**Figure 1D**).

### 2.3 Biospecimen collection and sample preparation

Patients enrolled in the DBBR consent to biospecimen collection with permission to link biospecimens with epidemiological and clinical data for the purpose of research. Upon enrollment in the DBBR, patients are scheduled to have blood samples drawn by phlebotomy service at Roswell Park. Blood samples are labeled with Roswell Park generated barcodes and immediately sent to the DBBR laboratory through the pneumatic tube system for processing and storage. Blood samples are processed into plasma, red blood cells, buffy coat, and serum and aliquoted into 0.5 mL straws by CryoBio System MAPI (Paris, France). Aliquots are then cryopreserved in liquid nitrogen. The time from biospecimen collection to storage is one hour or less to maintain biospecimen integrity (27).

#### 2.3.1 Targeted metabolomics

Banked serum samples were prepared and analyzed using the MxP Quant 500 kit (Biocrates Life Sciences AG, Innsbruck, Austria) in the Bioanalytics, Metabolomics, and Pharmacokinetics Shared Resource at Roswell Park accordance with the user manual. This assay has a high level of technical reproducibility and internal standards to ameliorate batch effects. 10μL of each supernatant, quality control samples, blank, zero sample, or calibration standard were added on the filterspot (already containing internal standard) in the appropriate wells of the 96-well plate. The plate was then dried under a gentle stream of nitrogen. Samples were derivatized with phenyl isothiocyanate for the amino acids and biogenic amines and dried again. Sample extract elution was performed with 5mM ammonium acetate in methanol. Sample extracts were diluted with either water for the HPLC-MS/MS analysis (1:1) or kit running solvent for flow injection analysis-MS/MS (50:1) using a Sciex 5500 mass spectrometer, which can measure up to 630 metabolites spanning 26 biochemical classes. Data were processed using MetIDQ software (Biocrates Life Sciences AG, vsn Oxygen-DB110-3005), and values below detection level were imputed using 0.1 times the smallest value in each metabolite; 25 metabolites were excluded due to >80% of samples being below the limit of detection.

#### 2.3.2 Cytokines

Cytokine profiles including adiponectin, leptin (HADK2MAG-61K-01), resistin (HADK1MAG-61K-02), and Th1/Th2 response cytokines (i.e., GM-CSF, IFN-γ, TNF-α, IL-1β, IL-2, IL-4, IL-5, IL-6, IL-12p70, IL-13, and IL-18) were assayed in the Flow and Immune Analysis Shared Resource at Roswell Park using commercially available kits from Millipore Sigma (Burlington, Massachusetts). The experiment and instrument set-up were performed based on the manufacturer’s kit instructions and data was acquired on a Luminex 200 instrument (Luminex Corporation, Austin, Texas). Analyte concentrations were determined by extrapolating individual experimental fluorescence intensity values against each analyte’s standard curve using the BeadView multiplex data analysis software, version 1.0 (Upstate Cell Signaling Solutions, Lake Placid, New York).

### 2.4 Statistical Analysis

In primary analyses, to identify differentially abundant circulating metabolites and cytokines according to body composition phenotype in EOC overall and in HGSOC, Limma moderated T-tests, (vsn 3.56.2) (28) adjusted for age at diagnosis, stage at diagnosis, and surgical debulking status were used. Limma moderated T-tests employ empirical Bayes methods to compare multiple metabolites or cytokines concurrently reducing variance and thus generating reliable results even when sample sizes are small (28). Covariates were selected *a priori* based on the extant literature demonstrating that age at diagnosis (29), stage at diagnosis (29), and surgical debulking status (30) are the only well-established prognostic factors for EOC. We examined the overlap in differentially abundant metabolites and cytokines in EOC overall and HGSOC using Venn diagrams. In exploratory analyses, we examined the interrelations of circulating serum cytokines and metabolites using Spearman correlations. Limma moderated T-tests for differential abundance analysis were performed using the “EnhancedVolcano” package and Spearman correlations were performed using the “mtcars” package in R (3.6.1), and descriptive characteristics were generated using SAS version 9.4.

## 3 Results

The epidemiological and clinical characteristics of the EOC study population are summarized in **Table 1**. The mean age at EOC diagnosis was 62.5 (11.2) years; most patients were diagnosed with advanced-stage disease (58.5%) and HGSOC tumors (62.1%). Additionally, most patients were overweight or obese according to BMI≥25 kg/m^2^ (75.4%) and 32.3% of patients were classified as low SMI, a proxy for sarcopenia. Further, 16.9% of patients had a Fit phenotype (**Figure 1A**), 50.8% had an Overweight/Obese phenotype (**Figure 1B**), 15.4% had a Sarcopenia/Obese phenotype (**Figure 1C**), and 16.9% had a Sarcopenia/Cachexia phenotype (**Figure 1D**).

**Table 1:** Clinical and epidemiological characteristics of the epithelial ovarian cancer study population^1^.

Clinical & Epidemiological Characteristics	M (SD) or N^2^ (%)
**Age at Diagnosis**	62.5 (± 11.2)
Self-Identified Race	
*White*	186 (95.4%)
*Black*	3 (1.5%)
*Other*	6 (3.1%)
Tumor Stage at Diagnosis	
*I*	28 (14.4%)
*II*	24 (12.3%)
*III*	79 (40.5%)
*IV*	35 (18.0%)
*Unknown*	29 (14.9%)
Histotype	
*High-Grade Serous*	121 (62.1%)
*Low-Grade Serous*	8 (4.1%)
*Clear Cell*	12 (6.2%)
*Endometrioid*	9 (4.6%)
*Mucinous*	5 (2.6%)
*Mixed*	32 (16.4%)
*Other*	8 (4.1%)
Surgical Debulking Status	
*Complete (R0)*	32 (16.4%)
*Optimal (≤1cm)*	91 (46.7%)
*Suboptimal (>1cm)*	27 (13.9%)
*Unknown*	45 (23.1%)
Smoking Status	
*Current*	32 (16.4%)
*Former*	58 (29.7%)
*Never*	104 (53.3%)
*Unknown*	1 (0.5%)
Body Mass Index (BMI)	
*Underweight (BMI<18.5 kg/m^2^)*	1 (0.5%)
*Normal Weight (18.5-24.9 kg/m^2^)*	47 (24.1%)
*Overweight (25-29.9 kg/m^2^)*	69 (35.4%)
*Obese (≥30 kg/m^2^)*	78 (40.0%)
Skeletal Muscle Index (SMI)^3^	
*Low Muscle (SMI<38.5 cm^2^/m^2^)*	63 (32.3%)
*Normal Muscle (SMI≥38.5 cm^2^/m^2^)*	132 (67.7%)
Body Composition Phenotype	
*Fit Phenotype*	33 (16.9%)
*Overweight/Obese Phenotype*	99 (50.8%)
*Sarcopenia/Obese Phenotype*	30 (15.4%)
*Sarcopenia/Cachexia Phenotype*	33 (16.9%)

^1^Patients included were enrolled in both the Data Bank and BioRepository and the Body Composition and Epithelial Ovarian Cancer Survival Study at Roswell Park Comprehensive Cancer Center.

^2^Columns may not sum to total N due to missing data.

^3^Skeletal muscle index (SMI) representing the ratio of muscle area in cm^2^ to height in m^2^, a proxy for sarcopenia.

### 3.1 Epithelial ovarian cancer overall

**Figure 2:**
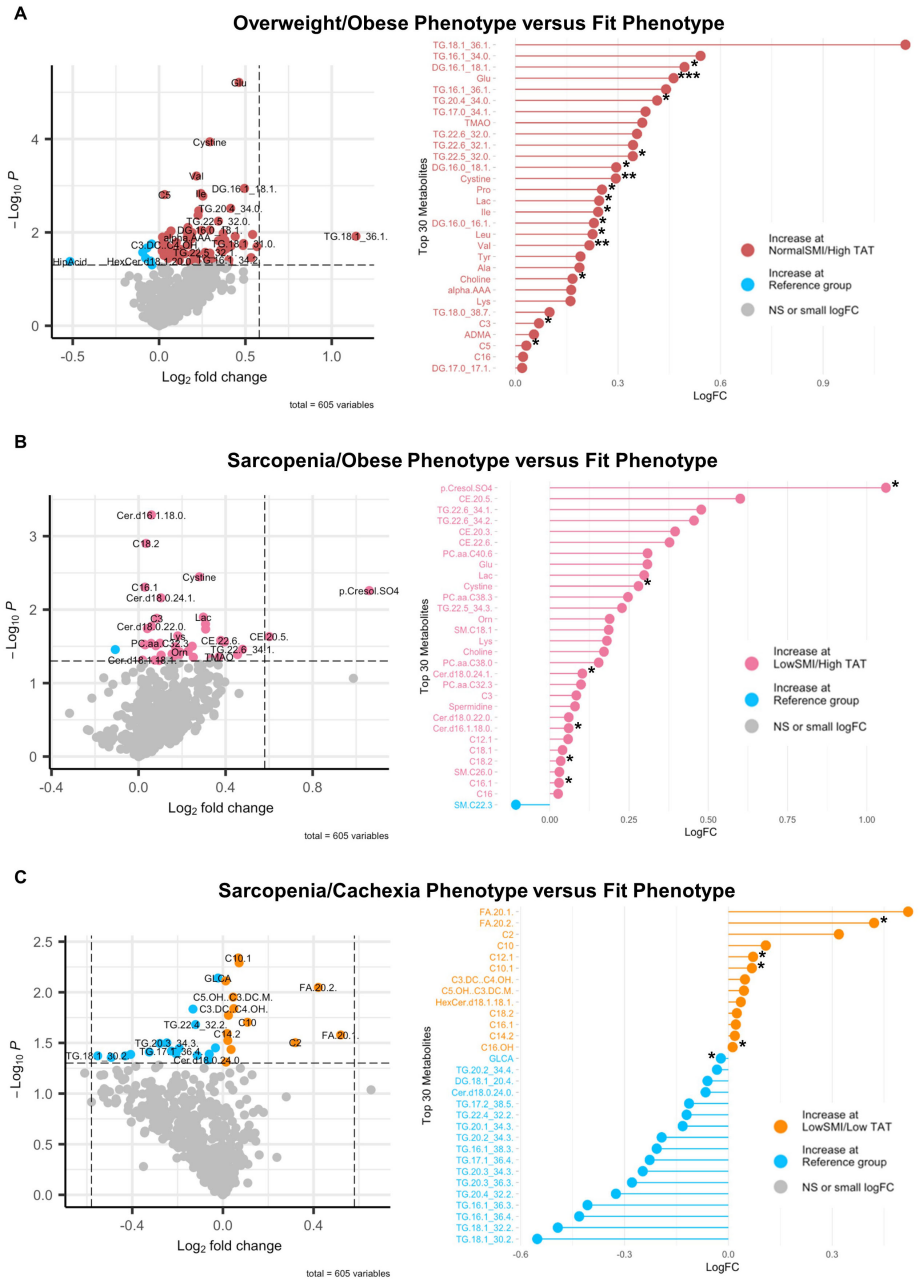
Volcano and lollipop plots depicting significant differentially abundant serum metabolites according to body composition phenotypes in EOC overall. Shown in the left panel are significant differentially abundant metabolites in the **(A)** normal SMI/high TAT (overweight/obese phenotype), **(B)** low SMI/high TAT (sarcopenia/obese phenotype), and **(C)** low SMI/low TAT (sarcopenia/cachexia phenotype) versus the reference/fit phenotype (normal SMI/low TAT). Red dots represent metabolites that are higher in the overweight/obese phenotype, pink dots represent metabolites that are higher in the sarcopenia/obese phenotype, orange dots represent metabolites that are higher in the sarcopenia/cachexia phenotype, and blue dots represent metabolites that are higher in the fit phenotype while blue dots represent metabolites that are higher in the fit phenotype. The right panel includes a lollipop chart showing the magnitude and direction of the top significantly differentially abundant metabolites in greater detail. SMI, skeletal muscle index wherein low SMI is a proxy for sarcopenia; TAT, total adipose tissue cross sectional area at L3; NS, non-significant. Differentially abundant metabolites are significant at p<0.05 unless indicated otherwise: *p<0.01; **p<0.001; ***p<0.0001.

To define associations of body composition with targeted metabolomics in EOC overall, we conducted differential metabolite abundance analyses comparing the Fit phenotype to three at-risk phenotypes (Overweight/Obese, Sarcopenia/Obese, and Sarcopenia/Cachexia) in the overall study population (**Figure 2**). For each comparison, the volcano plot (left panel) provides an overall summary of differentially abundant metabolites between the Fit and at-risk phenotypes and a more detailed lollipop plot (right panel) shows the magnitude and direction of the most significantly differentially abundant metabolites.

First, in comparing the Overweight/Obese versus the Fit phenotype (**Figure 2A**), we noted significantly increased abundance of several triacylglycerides and diacylglycerides, numerous amino acids including glutamate, branched-chain amino acids (i.e., leucine, isoleucine, and valine), proline, tyrosine, lysine, and alanine, trimethylamine N-oxide (TMAO), cystine, lactic acid, asymmetric dimethylarginine (ADMA), α-aminoadipic acid, and three acylcarnitines, with the most notable being propionylcarnitine. Next, in comparing the Sarcopenia/Obese versus the Fit phenotype (**Figure 2B**), we observed significant increased abundance of p-Cresol sulfate, triacylglycerides, cholesteryl esters, ceramides, phosphatidylcholines, sphingomyelins, amino acids (most notably glutamate), amino acid related metabolites (i.e., ornithine and cystine), lactic acid, choline, spermidine, and propionylcarnitine and several long-chain acylcarnitines and significantly decreased abundance of sphingomyelin C22:3. Last, in comparing the Sarcopenia/Cachexia versus the Fit phenotype (**Figure 2C**), we observed significant increased abundance of eicosenoic and eicosadienoic acid, a hexosylceramide, and several acylcarnitines (e.g., short-, medium-, and long-chain) and decreased abundance of glycolithocholic acid (GLCA), several triacylglycerides, one diacylglyceride, and one ceramide.

**Figure 3:**
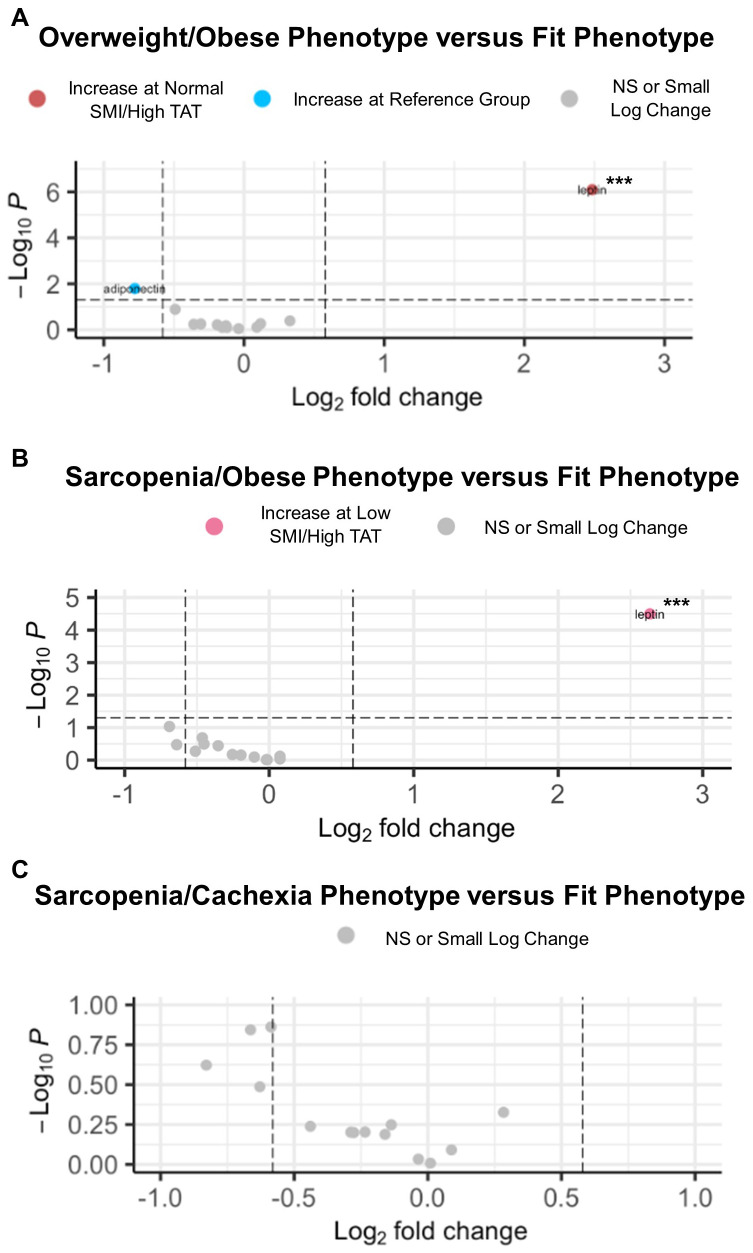
Volcano plots depicting significant differentially abundant serum cytokines according to body composition phenotypes in EOC overall. **(A)** Significant differentially abundant metabolites in the normal SMI/high TAT (overweight/obese) phenotype versus the reference/fit phenotype (normal SMI/low TAT). **(B)** Significant differentially abundant metabolites in the low SMI/high TAT (sarcopenia/obese) phenotype versus the reference/fit phenotype. **(C)** Significant differentially abundant metabolites in the low SMI/low TAT (sarcopenia/cachexia) phenotype versus the reference/fit phenotype. SMI, skeletal muscle index wherein low SMI is a proxy for sarcopenia; TAT, total adipose tissue cross sectional area at L3; NS, non-significant. Differentially abundant metabolites are significant at p<0.05 unless indicated otherwise: *p<0.01; **p<0.001; ***p<0.0001.

Next, circulating cytokine abundance according to joint-exposure muscle and adiposity body composition phenotypes was assessed in EOC patients overall (**Figure 3**). In comparing the Overweight/Obese versus the Fit phenotype, we observed significantly increased abundance of leptin (**Figure 3A**). For patients with the Sarcopenia/Obese phenotype versus Fit phenotype, we noted significantly increased abundance of leptin and significantly decreased abundance of adiponectin (**Figure 3B**). However, no significant differentially abundant cytokines were observed in comparisons between patients with the Sarcopenia/Cachexia phenotype versus the Fit phenotype (**Figure 3C**).

### 3.2 High-grade serous ovarian carcinoma

**Figure 4:**
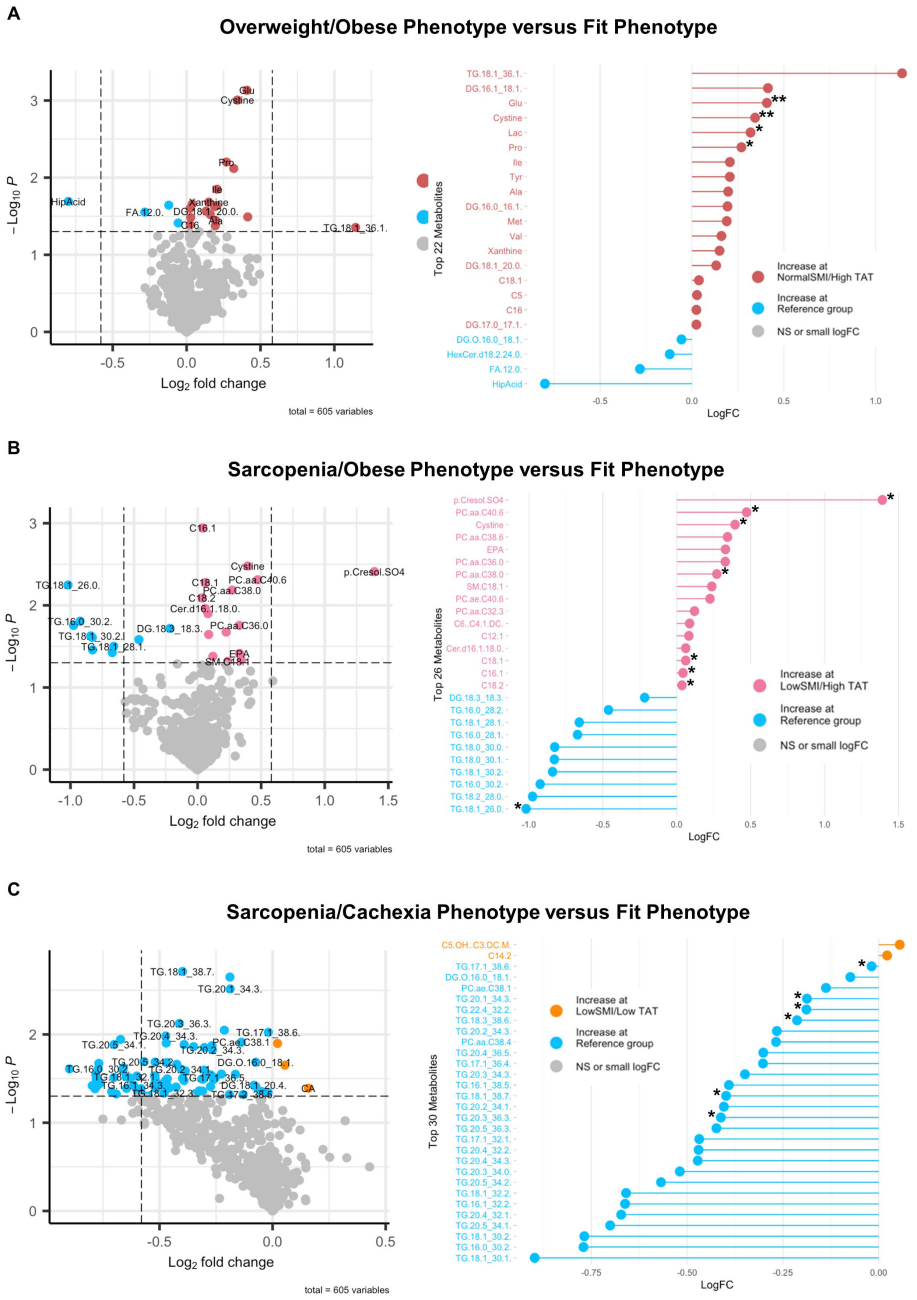
Volcano and lollipop plots depicting significant differentially abundant serum metabolites according to body composition phenotypes in HGSOC. Shown in the left panel are significant differentially abundant metabolites in the **(A)** normal SMI/high TAT (overweight/obese phenotype), **(B)** low SMI/high TAT (sarcopenia/obese phenotype), and **(C)** low SMI/low TAT (sarcopenia/cachexia phenotype) versus the reference/fit phenotype (normal SMI/low TAT). Red dots represent metabolites that are higher in the overweight/obese phenotype, pink dots represent metabolites that are higher in the sarcopenia/obese phenotype, orange dots represent metabolites that are higher in the sarcopenia/cachexia phenotype, and blue dots represent metabolites that are higher in the fit phenotype while blue dots represent metabolites that are higher in the fit phenotype. The right panel includes a lollipop chart showing the magnitude and direction of the top significantly differentially abundant metabolites in greater detail. SMI, skeletal muscle index wherein low SMI is a proxy for sarcopenia; TAT, total adipose tissue cross sectional area at L3; NS, non-significant. Differentially abundant metabolites are significant at p<0.05 unless indicated otherwise: *p<0.01; **p<0.001; ***p<0.0001.

**Figure 5:**
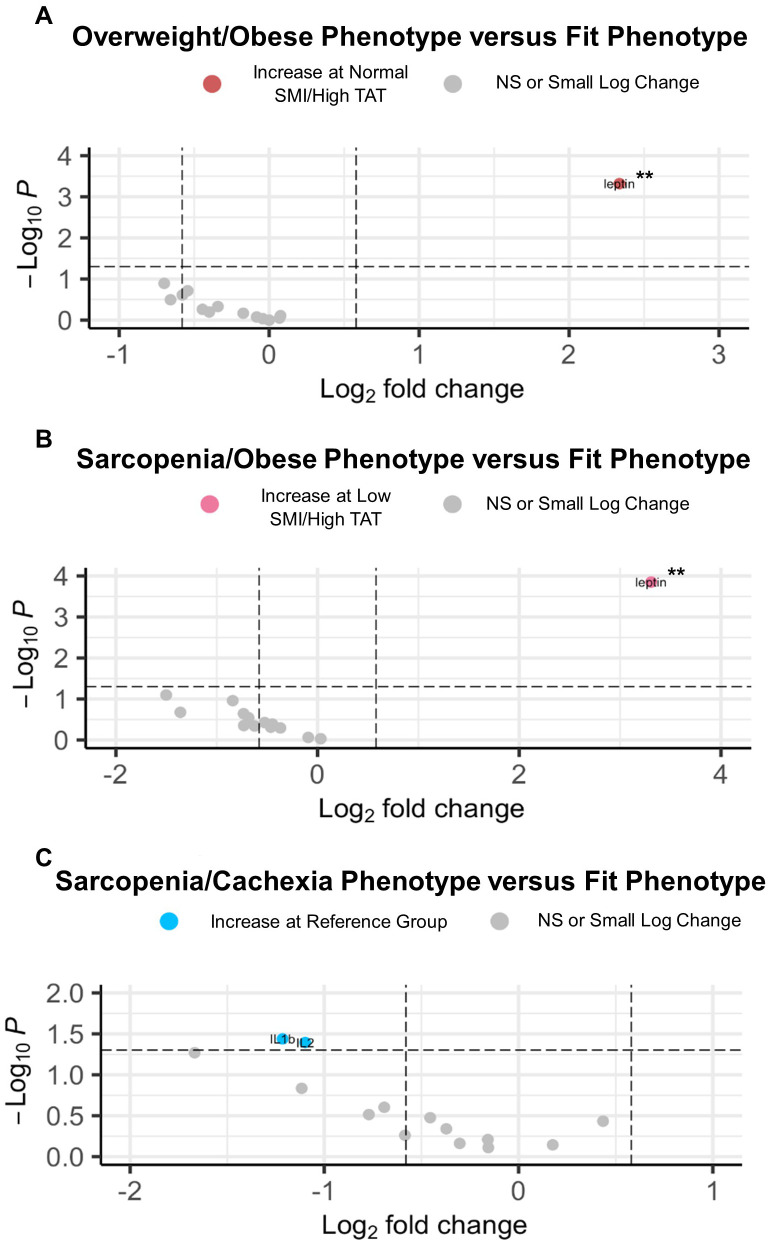
Volcano plots depicting significant differentially abundant serum cytokines according to body composition phenotypes in HGSOC. **(A)** Significant differentially abundant metabolites in the normal SMI/high TAT (overweight/obese) phenotype versus the reference/fit phenotype (normal SMI/low TAT). **(B)** Significant differentially abundant metabolites in the low SMI/high TAT (sarcopenia/obese) phenotype versus the reference/fit phenotype. **(C)** Significant differentially abundant metabolites in the low SMI/low TAT (sarcopenia/cachexia) phenotype versus the reference/fit phenotype. SMI, skeletal muscle index wherein low SMI is a proxy for sarcopenia; TAT, total adipose tissue cross sectional area at L3; NS, non-significant. Differentially abundant metabolites are significant at p<0.05 unless indicated otherwise: *p<0.01; **p<0.001; ***p<0.0001.

Associations of body composition phenotype with targeted metabolomics in HGSOC are presented in **Figure 4**. In comparing the Overweight/Obese phenotype versus the Fit phenotype (**Figure 4A**), we noted significantly increased abundance of a triacylglyceride, several diacylglycerides, several amino acids including glutamate and branched chain amino acids (i.e., leucine, isoleucine, and valine), lactic acid, xanthine, and three acylcarnitines including valerylcarnitine, octadecenoylcarnitine, and hexadecanoylcarnitine, and significantly decreased abundance of one diacylglyceride, one hexosylceramide, lauric acid, and hippuric acid. For the Sarcopenia/Obese phenotype versus the Fit phenotype (**Figure 4B**), we observed increased abundance of p-Cresol sulfate, several phosphatidylcholines, cystine, eicosapentaenoic acid, one sphingomyelin, one ceramide, and several acylcarnitines including dodecenoylcarnitine, hexadecenoylcarnitine, cctadecenoylcarnitine, and octadecadienylcarnitine, and decreased abundance of several triacylglycerides and one diacylglyceride. Lastly, in comparing the Sarcopenia/Cachexia phenotype versus the Fit phenotype (**Figure 4C**) we observed significantly increased abundance of circulating hydroxyvalerylcarnitine and tetradecadienoylcarnitine and significantly decreased abundance of several triacylglycerides, one diacylglyceride, and two phosphatidylcholines. The associations of body composition phenotypes with cytokines in HGSOC are presented in **Figure 5**. In comparison with the Fit phenotype, patients with the Overweight/Obese (**Figure 5A**) and Sarcopenia/Obese phenotypes (**Figure 5B**) had significantly increased abundance of leptin. Conversely, in patients with the Sarcopenia/Cachexia phenotype, we observed significantly decreased abundance of IL-1β and IL-2 (**Figure 5C**).

### 3.3 Comparison of EOC overall and HGSOC

**Figure 6:**
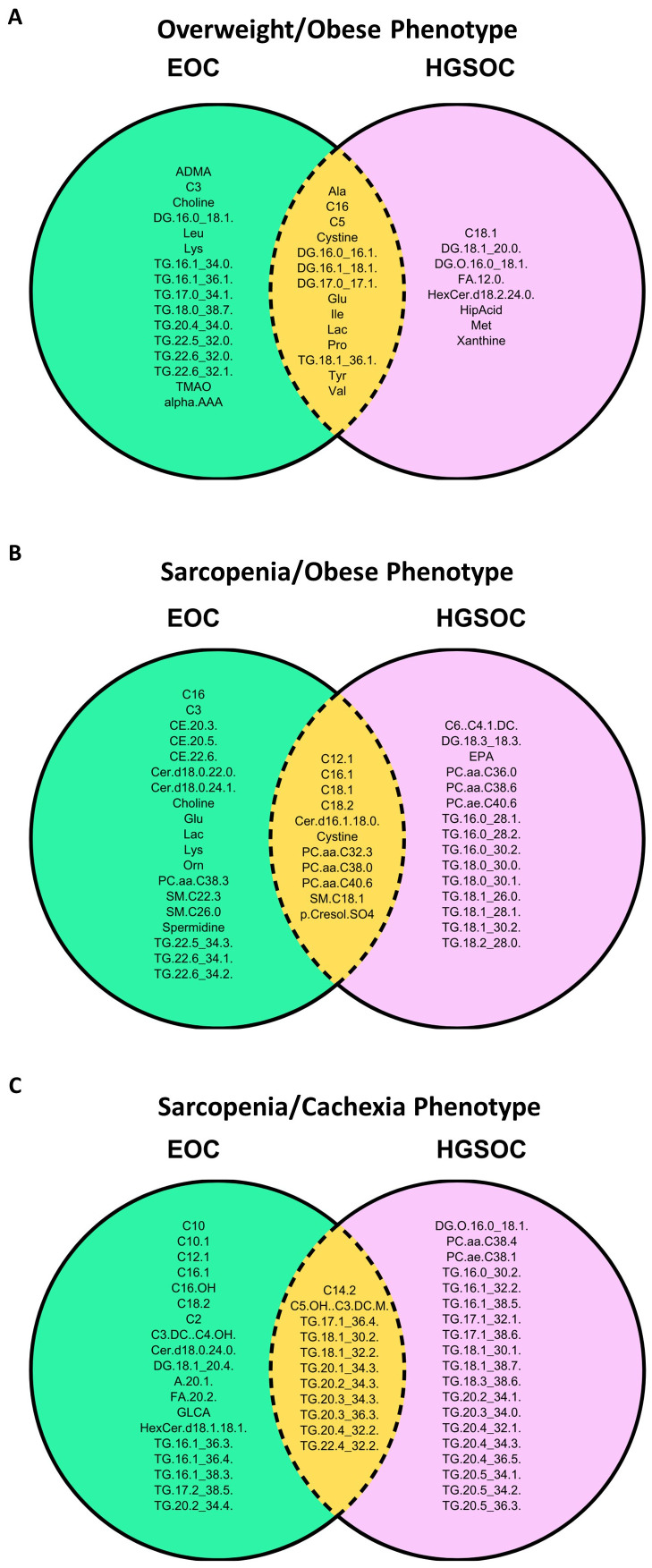
Venn diagrams depicting the significant differentially abundant serum metabolites and cytokines that were similar and different between EOC overall and HGSOC according to the **(A)** Overweight/Obese phenotype (normal SMI/high TAT), **(B)** Sarcopenia/Obese phenotype (low SMI/high TAT), and **(C)** Sarcopenia/Cachexia phenotype (low SMI/low TAT) versus the fit phenotype (normal SMI/low TAT). SMI, skeletal muscle index wherein low SMI is a proxy for sarcopenia; TAT, total adipose tissue cross sectional area at L3.

We examined the overlap in differentially abundant immuno-metabolic biomarkers between EOC overall and HGSOC for each body composition phenotype in **Figure 6**. In patients with the Overweight/Obese phenotype (**Figure 6A**), we noted that several amino acids (most notably glutamate), branched-chain amino acids (isoleucine and valine), lactic acid, valerylcarnitine, hexadecanoylcarnitine, several diacylglycerides, and one triacylglyceride were similar in EOC overall and HGSOC. In patients with the Sarcopenia/Obese phenotype (**Figure 6B**), we noted that several long-chain acylcarnitines, phosphatidylcholines, a ceramide, a sphingomyelin, cystine, and p-Cresol sulfate were increased in abundance for EOC overall and HGSOC. Finally, in patients with the Sarcopenia/Cachexia phenotype (**Figure 6C**), we noted that hydroxyvalerylcarnitine, tetradecadienoylcarnitine, and several triacylglycerides were increased in patients with EOC overall and HGSOC.

### 3.4 Exploratory analyses

Finally, in exploratory analyses, we observed an inverse relationship between several Th1 cytokines and metabolites with increased abundance in the at-risk phenotypes in EOC overall (**Supplementary Figure 1**) and HGSOC (**Supplementary Figure 2**). We also observed positive correlations between Th2 cytokines and metabolites with increased abundance in the at-risk phenotypes in EOC overall (**Supplementary Figure 1**) and HGSOC (**Supplementary Figure 2**).

## 4 Discussion

We previously reported that three at-risk body composition phenotypes before chemotherapy (Overweight/Obese, Sarcopenia/Cachexia, and Sarcopenia/Obese) are associated with dramatic (up to twofold) increases in mortality in EOC patients in the BComES Study (22, 26). In the current investigation of the associations of body composition phenotypes with immuno-metabolic biomarkers, we show that clinically relevant high-adiposity and low-muscle phenotypes are biologically distinct phenotypes with unique circulating cytokine and metabolic milieus.

For instance, patients with the Overweight/Obese phenotype had increased abundance of known markers of excess adiposity (i.e., triacylglycerides, glutamate, branch-chain amino acids, TMAO, α-aminoadipic acid, lactic acid, ADMA, and leptin) (31–38) while patients with the Sarcopenia/Obese phenotype had increased abundance of markers that suggested both excess adiposity (i.e., triacylglycerides, glutamate, phosphatidylcholines, ceramides, and cholesteryl esters) (31, 32, 39–41) and low muscle/muscle wasting (i.e., phosphatidylcholines, ceramides, and long-chain acylcarnitines) (42–46) via their impact on insulin resistance (39, 42, 45). Interestingly, there were also differences in adiposity markers between these two phenotypes. For example, there was greater abundance of phosphatidylcholines and ceramides in Sarcopenia/Obese phenotype, but not in the Overweight/Obese phenotype. While phosphatidylcholines and ceramides are markers of excess adiposity (39–41) they are also markers of insulin resistance (39, 42) which is known to contribute to muscle loss/wasting (42–46). Moreover, in the Sarcopenia/Cachexia phenotype we observed greater abundance of markers suggestive of low muscle/muscle wasting (43–46) and low adiposity (47) in comparison to the Fit phenotype. While the Sarcopenia/Cachexia and Fit phenotypes both have low adiposity, distinct immuno-metabolic biomarkers emerged when the two were compared that revealed potentially immune suppressive markers in the Sarcopenia/Cachexia phenotype that were not present in the Fit phenotype.

The previously reported differences in survival according to joint-exposure muscle and adiposity body composition phenotypes (26) and the differences in the immuno-metabolic milieus reported herein confirms the need to consider skeletal muscle mass when examining the impact of excess adiposity in clinical oncology and cancer epidemiological studies. Failing to jointly consider adiposity and skeletal muscle body composition phenotypes may attenuate or eliminate important clinically and biologically relevant relationships in cancer patient populations.

To this end, we noted several of the metabolites that were significantly higher in the three at-risk phenotypes (Overweight/Obese, Sarcopenia/Cachexia, and Sarcopenia/Obese) in EOC overall and HGSOC have also been implicated in immune suppression (48–60) and tumor progression (61–67). For instance, in patients with high-adiposity phenotypes versus the Fit phenotype, triacylglycerides and diacylglycerides (54–60), lactic acid (48) and phosphatidylcholines (49) were all significantly elevated and these metabolites have been shown to promote regulatory T cell, myeloid-derived suppressor cell, and M2-like macrophage populations. Additionally, cholesteryl esters, increased in the Sarcopenia/Obese phenotype, have been associated with inhibition of CD8+ T cell populations (50). Excess methionine (51) and branched-chain amino acids (i.e., leucine, isoleucine, and valine) (52), increased in high-adiposity phenotypes, may also contribute to a blunted anti-tumor immune response and be linked to dysfunction in tumor-infiltrating T cells. Further, higher leptin and lower adiponectin, observed in the high-adiposity phenotypes, play important roles in immune suppression and tumor progression (68–71). Finally, long-chain acylcarnitines, which were increased in all at-risk body composition phenotypes, may contribute to T cell exhaustion and dysfunction (53). Further, evidence in our data demonstrated that several metabolites with greater abundance in the at-risk phenotypes were negatively correlated with Th1 cytokines and positively correlated with Th2 cytokines further supporting the potential role of immune suppression and tumor progression in these at-risk body composition phenotypes.

Importantly, in comparison to women with the Fit phenotype, women with the Overweight/Obese, Sarcopenia/Obese, and Sarcopenia/Cachexia phenotypes all had increased abundance of long-chain acylcarnitines, a transporter of long-chain fatty acids for β-oxidation (53). Considering the potential role of long-chain acylcarnitines in immune suppression (53), patients with these body composition phenotypes may derive benefit from therapeutically targeting acylcarnitines. While we acknowledge the pleiotropic effects of metformin on metabolism, metformin, which has been proven a safe and effective treatment for diabetes (72), has been found to decrease acylcarnitine levels (73) and is safe for use in cancer patients (74). Combined with lifestyle intervention ancillary to primary first-line therapy (75), metformin could be a feasible, cost-effective therapeutic target to address metabolic dysfunction in patients with at-risk body composition phenotypes with the potential to improve treatment response and outcomes in EOC overall and in HGSOC.

Not only did we observe a consistent suggestion that biomarkers associated with immune suppression were more abundant among high-adiposity and low-muscle phenotypes, but we also observed that patients with the Fit phenotype had increased abundance of metabolites and cytokines suggestive of immune activation and an anti-tumor response. For example, we observed increased abundance of lauric acid which is suggested to have antiproliferative and pro-apoptotic activity in cancer cells (76). Furthermore, increased abundance of Th1 and anti-tumor cytokines was noted in the Fit phenotype suggesting that these patients have increased presence of Th1 cells leading to increased cytotoxic T cell activation and improved anti-tumor immunity (77–79). Together, these findings suggest that a Fit body composition phenotype (normal SMI/low TAT) may contribute to improved anti-tumor response, potentially conferring improved treatment response in these patients. Indeed, our previous work has shown that among EOC patients receiving immunotherapy, patients with the Fit phenotype have significantly improved 5-year survival in comparison to patients with at-risk phenotypes (data not shown; p-value=0.04) (80). Considering that body composition is modifiable (81, 82), targeted exercise programs according to body composition phenotype could be harnessed to improve treatment response and outcomes in patients receiving immunotherapy.

### 4.1 Limitations

The BComES Study comprises a clinically and demographically homogeneous EOC patient population, potentially limiting the generalizability of these findings to more diverse patient populations. Additionally, we were not statistically powered to investigate the associations of body composition phenotype with immuno-metabolic biomarkers in less common EOC histotypes, including low-grade serous, endometrioid, mucinous, or clear cell tumors. Lastly, observations reported herein reflect the circulating immuno-metabolic milieu, which may not be reflective of the TIME, the most relevant site for tumor progression. However, previously published reports do suggest the circulating immuno-metabolic microenvironment is a representative proxy for the ovarian TIME (83, 84).

Important strengths of our pilot study include the relatively large, well-characterized EOC patient population with treatment naïve biospecimens linked with detailed clinical and epidemiological data, and the ability to account for objectively assessed adiposity and skeletal muscle in our analyses. Moreover, body composition and blood samples were collected in the peri-diagnosis period identifying a modifiable exposure that is implicated in immune suppression and tumor progression and could be targeted through lifestyle intervention ancillary to primary treatment. Additionally, we employed a novel approach to understand the impact of body composition on immuno-metabolic biomarkers by using targeted metabolomics and Luminex analyses according to joint muscle/adiposity body composition phenotypes, which has not previously explored in cancer patient populations.

### 4.2 Conclusions

We have identified four clinically significant body composition phenotypes known to predict survival in EOC and HGSOC which also have distinct circulating metabolic and cytokine milieus (26). In comparison to EOC patients with a Fit phenotype, patients with high-adiposity and low-muscle phenotypes have higher concentrations of metabolites and cytokines known to be associated with immune suppression and tumor progression. Conversely, patients with the Fit phenotype have higher concentrations of metabolites and cytokines indicative of immune activation and tumor suppression. Considering body composition is modifiable (85–87), these findings provide rationale for leveraging lifestyle intervention as a safe, feasible strategy for potentially improving response to standard-of-care chemotherapy and novel immunotherapies in patients diagnosed with a highly fatal malignancy with poor treatment response (75).

## Data Availability

The datasets presented in this article are not readily available because the data supporting the findings of this study are not publicly available but may be shared upon reasonable request in a de-identified format for use in meta-analysis or pooled analyses. All data requests should be addressed to the corresponding author and will be dependent upon an approved data sharing agreement with Roswell Park Comprehensive Cancer Center. Requests to access the datasets should be directed to Rikki Cannioto rikki.cannioto@roswellpark.org.
