# Supplementary material for: Clinically relevant body composition phenotypes are associated with distinct circulating cytokine and metabolomic milieus in epithelial ovarian cancer patients
Source: Front Immunol. 2024 Nov 7;15:1419257. doi: 10.3389/fimmu.2024.1419257 (PMC11578747; doi:10.3389/fimmu.2024.1419257)
Supplement: Supplementary file 1 [file DataSheet1.docx]

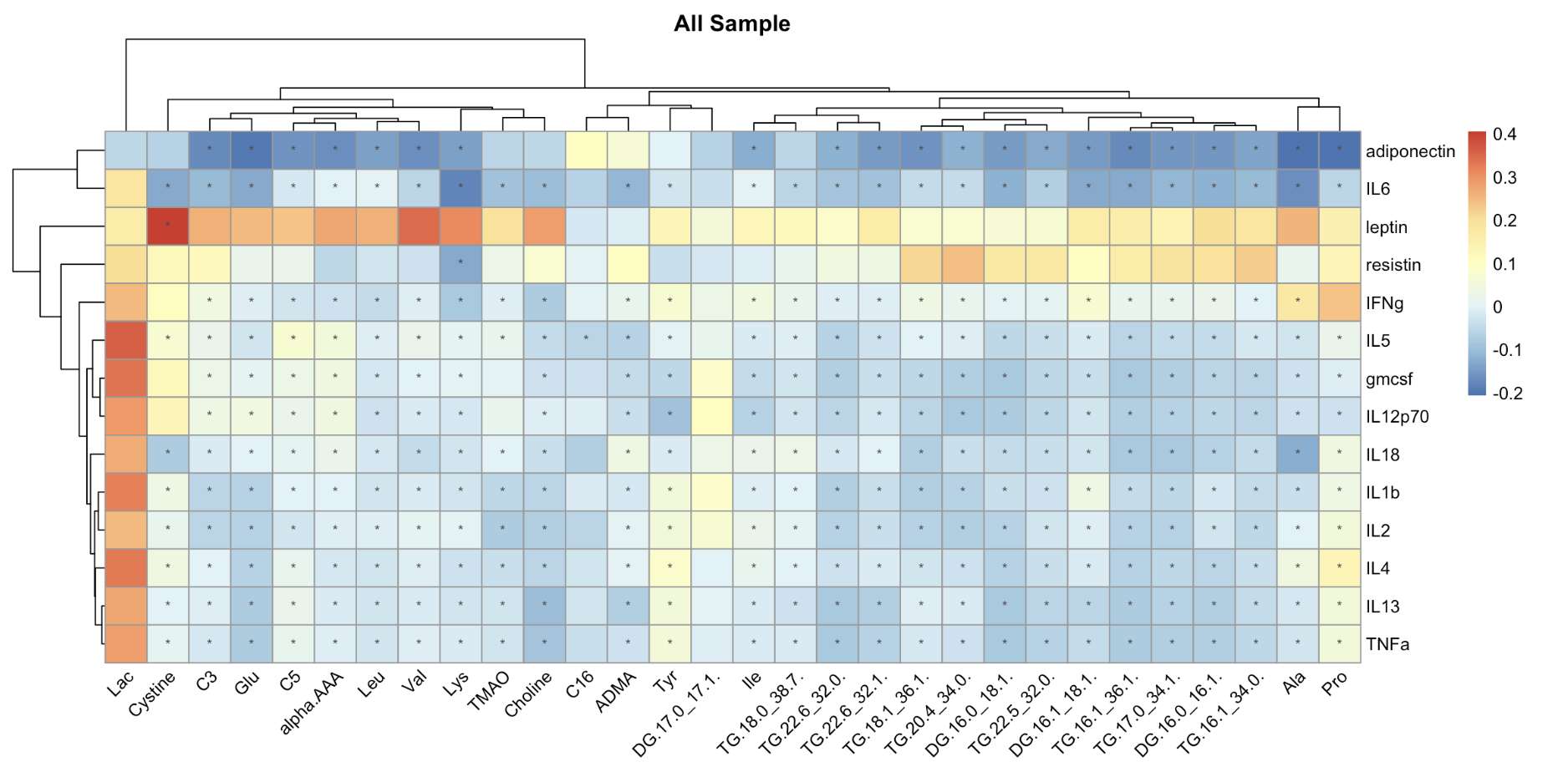


**A**

**Overweight/Obese Phenotype versus Fit Phenotype**


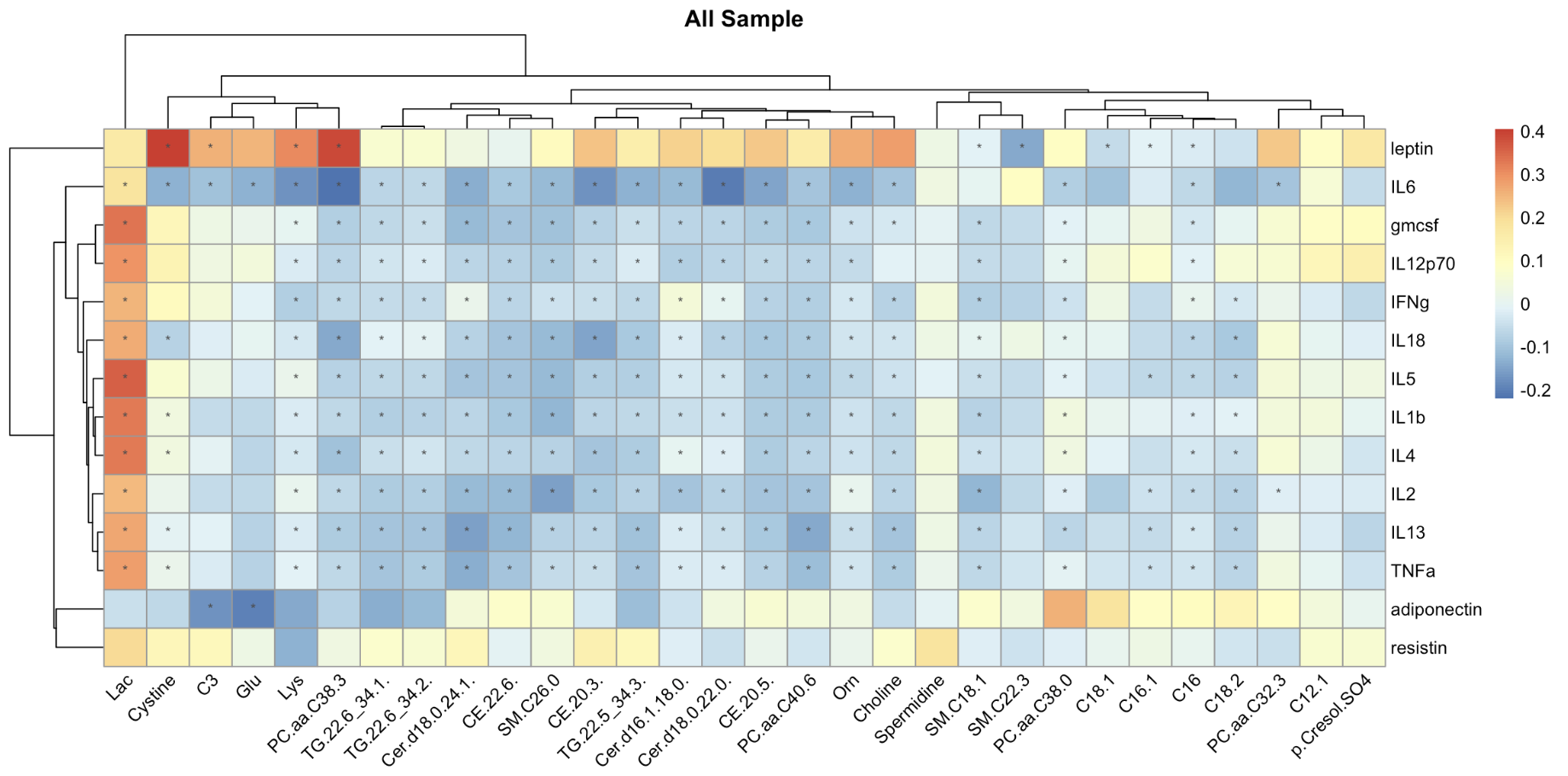


**B**

**Sarcopenia/Obese Phenotype versus Fit Phenotype**


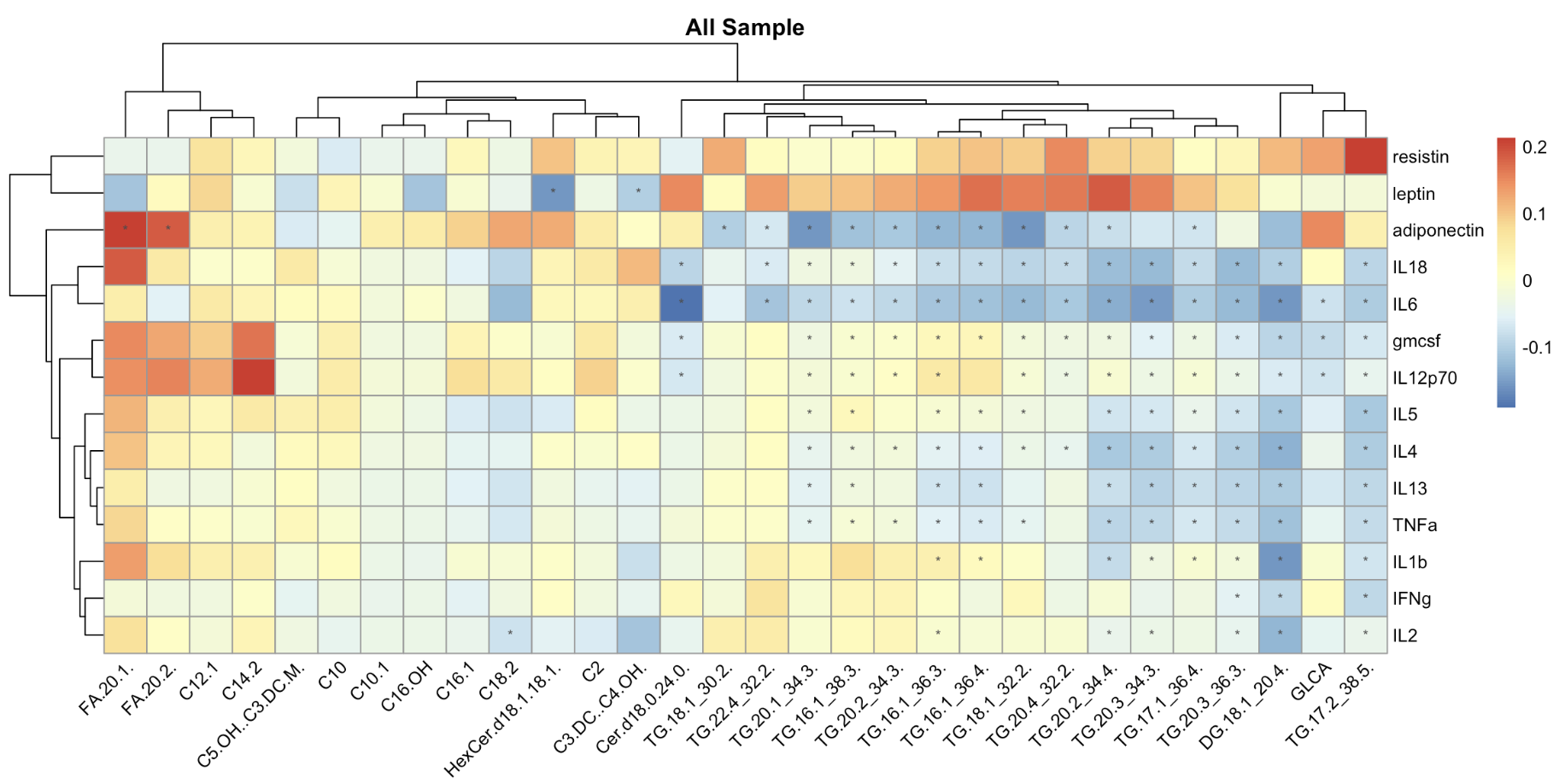


**C**

**Sarcopenia/Cachexia Phenotype versus Fit Phenotype**

**Supplemental Figure 1.** Heat maps showing the Spearman correlations of the top differentially abundant metabolites and cytokines in EOC patients overall with the **(A)** Overweight/Obese phenotype, **(B)** Sarcopenia/Obese phenotype; and **(C)** Sarcopenia/Cachexia phenotype.

**A**

**Overweight/Obese Phenotype versus Fit Phenotype**


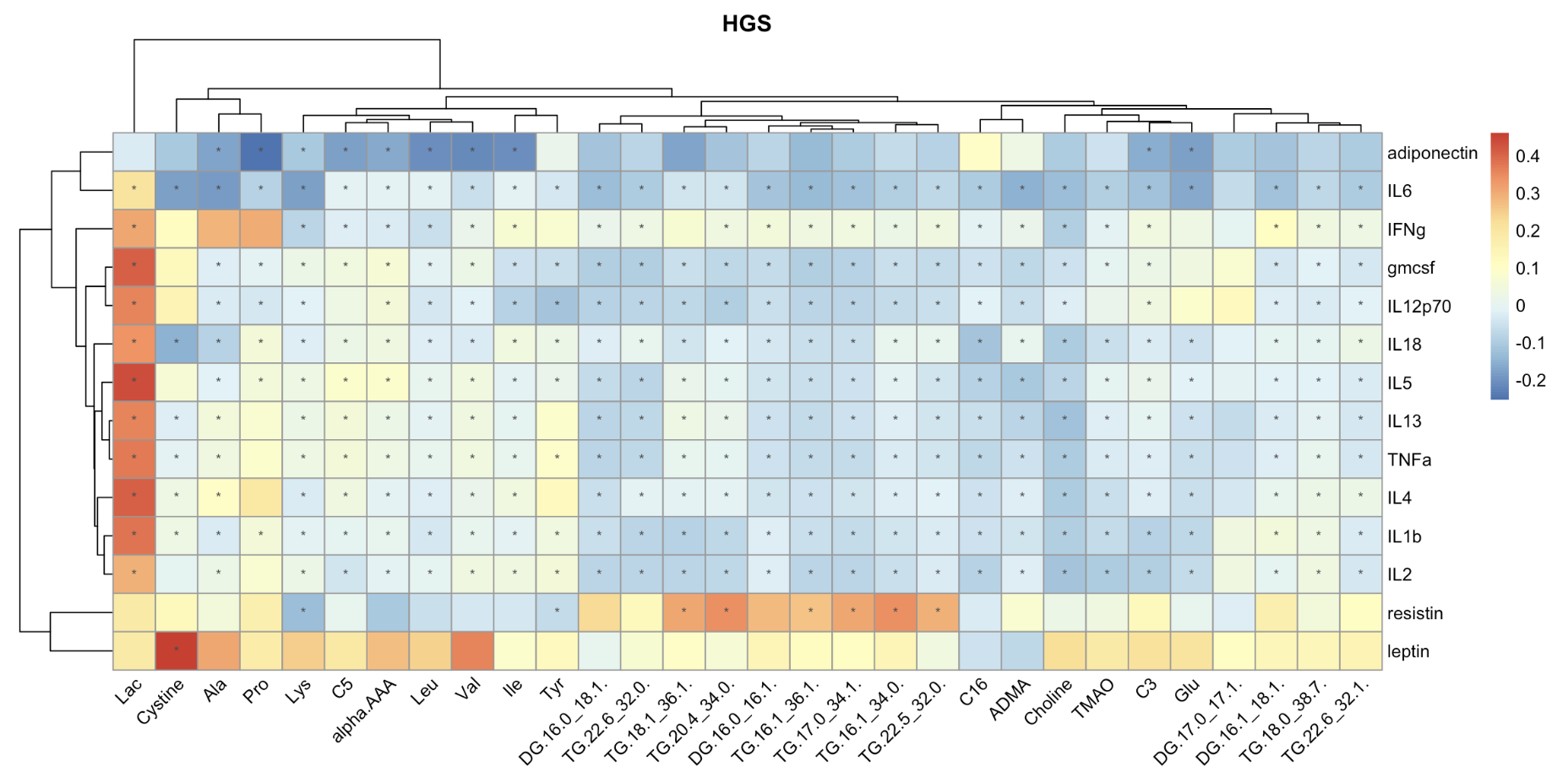


**B**

**Sarcopenia/Obese Phenotype versus Fit Phenotype**


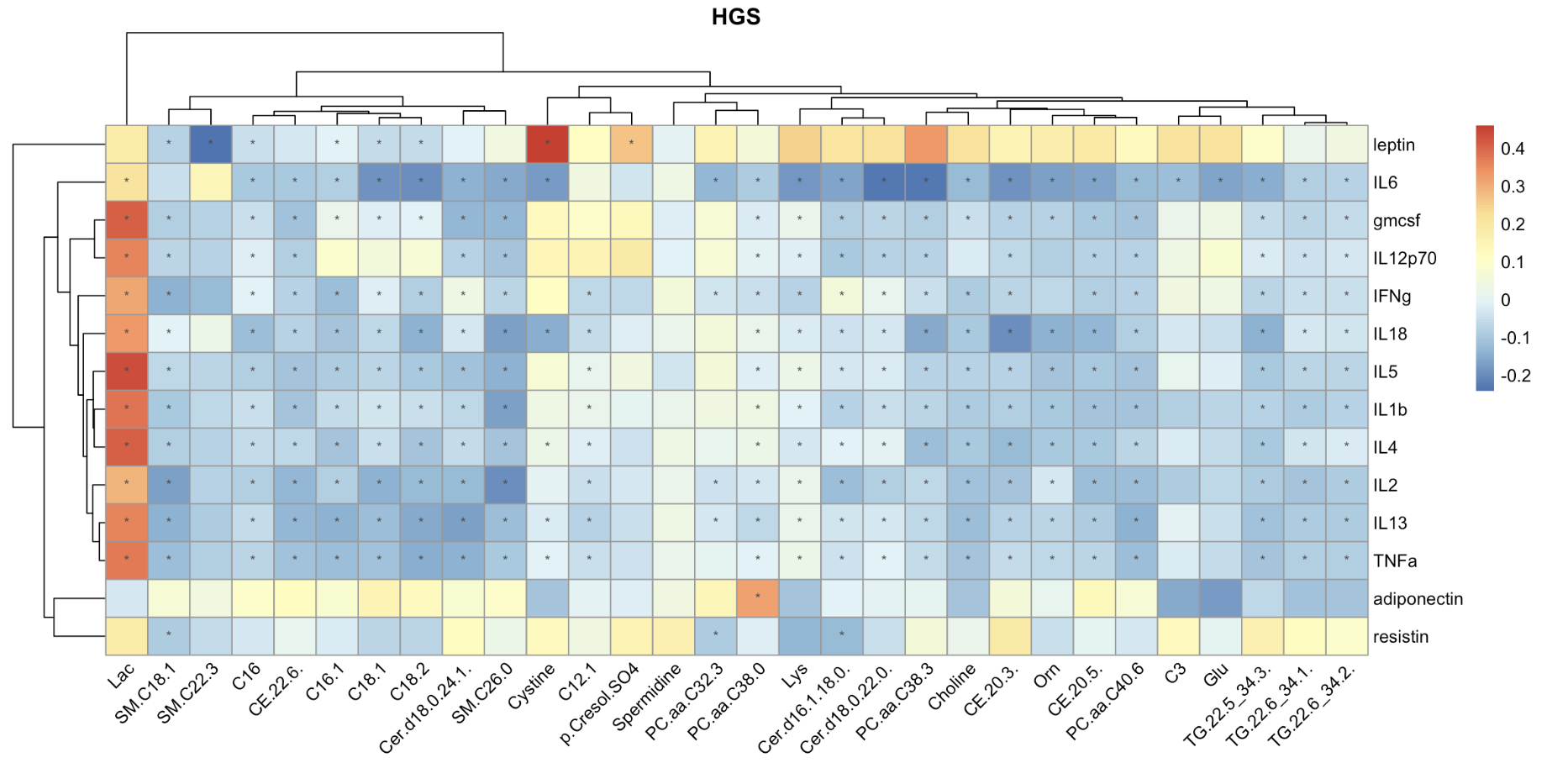


**C**


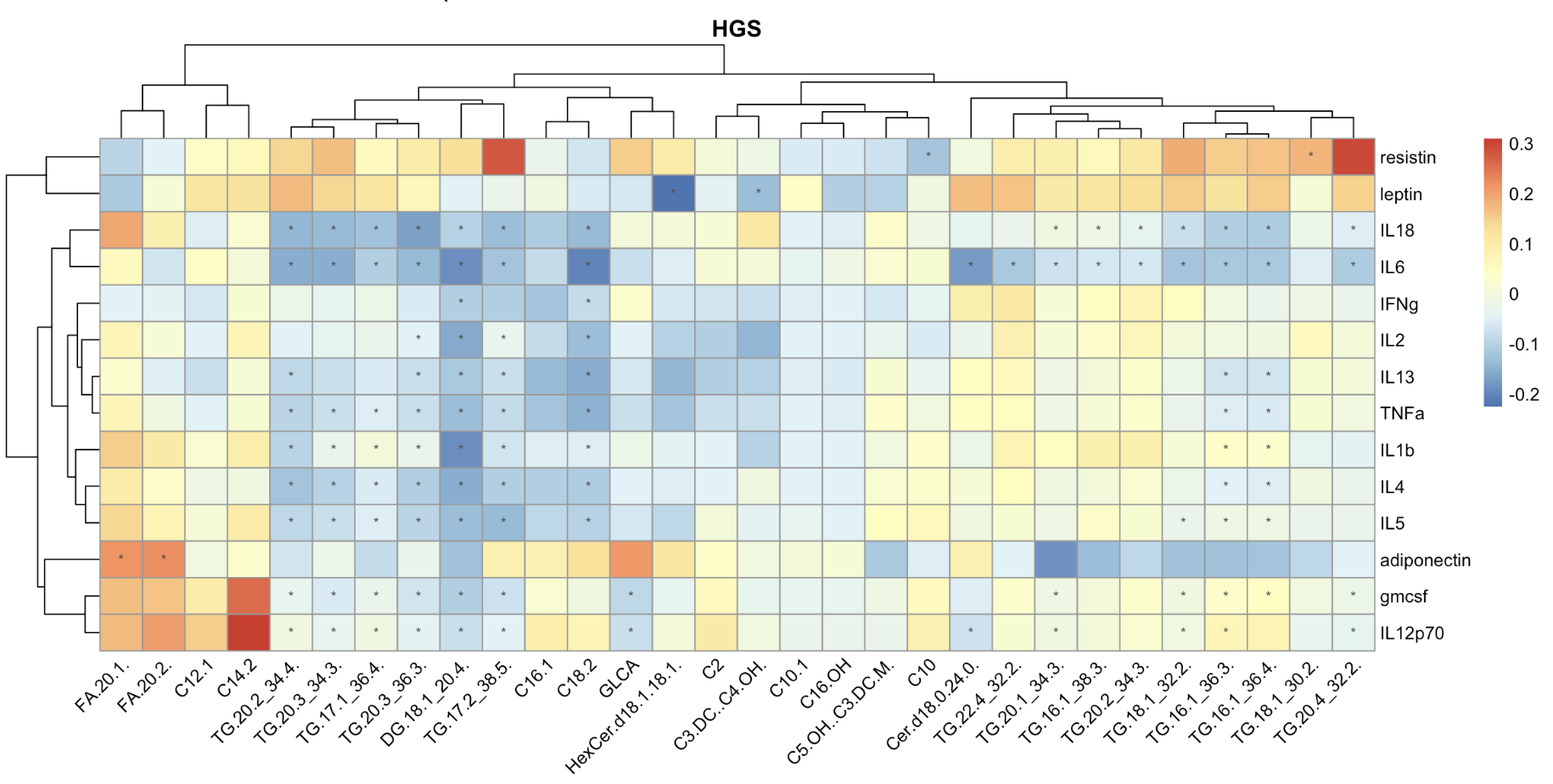


**Sarcopenia/Cachexia Phenotype versus Fit Phenotype**

**Supplemental Figure 2.** Heat maps showing the Spearman correlations of the top differentially abundant metabolites and cytokines in HGSOC with the **(A)** Overweight/Obese phenotype, **(B)** Sarcopenia/Obese phenotype; and **(C)** Sarcopenia/Cachexia phenotype.
